# Effects of Survival Motor Neuron Protein on Germ Cell Development in Mouse and Human

**DOI:** 10.3390/ijms22020661

**Published:** 2021-01-11

**Authors:** Wei-Fang Chang, Min Peng, Jing Hsu, Jie Xu, Huan-Chieh Cho, Hsiu-Mei Hsieh-Li, Ji-Long Liu, Chung-Hao Lu, Li-Ying Sung

**Affiliations:** 1 Institute of Biotechnology, National Taiwan University, Taipei 106, Taiwan; weifange@gmail.com (W.-F.C.); larel2105@gmail.com (M.P.); hsujing912@gmail.com (J.H.); 2 Center for Advanced Models for Translational Sciences and Therapeutics, University of Michigan Medical Center, Ann Arbor, MI 48109, USA; jiex@med.umich.edu; 3 Animal Resource Center, National Taiwan University, Taipei 106, Taiwan; cchaser2002@gmail.com; 4 Department of Life Science, National Taiwan Normal University, Taipei 116, Taiwan; hmhsieh@ntnu.edu.tw; 5 MRC Functional Genomics Unit, Department of Physiology, Anatomy and Genetics, University of Oxford, Oxford OX1 3PT, UK; jilong.liu@dpag.ox.ac.uk; 6 School of Life Science and Technology, ShanghaiTech University, Shanghai 201210, China; 7 Department of Obstetrics and Gynecology, Mackay Memorial Hospital, Taipei 105, Taiwan; 8 Agricultural Biotechnology Research Center, Academia Sinica, Taipei 115, Taiwan

**Keywords:** survival motor neuron, spermatogenesis, azoospermia

## Abstract

Survival motor neuron (SMN) is ubiquitously expressed in many cell types and its encoding gene, survival motor neuron 1 gene (SMN1), is highly conserved in various species. SMN is involved in the assembly of RNA spliceosomes, which are important for pre-mRNA splicing. A severe neurogenic disease, spinal muscular atrophy (SMA), is caused by the loss or mutation of SMN1 that specifically occurred in humans. We previously reported that SMN plays roles in stem cell biology in addition to its roles in neuron development. In this study, we investigated whether SMN can improve the propagation of spermatogonia stem cells (SSCs) and facilitate the spermatogenesis process. In in vitro culture, SSCs obtained from SMA model mice showed decreased growth rate accompanied by significantly reduced expression of spermatogonia marker promyelocytic leukemia zinc finger (PLZF) compared to those from heterozygous and wild-type littermates; whereas SMN overexpressed SSCs showed enhanced cell proliferation and improved potency. In vivo, the superior ability of homing and complete performance in differentiating progeny was shown in SMN overexpressed SSCs in host seminiferous tubule of transplant experiments compared to control groups. To gain insights into the roles of SMN in clinical infertility, we derived human induced pluripotent stem cells (hiPSCs) from azoospermia patients (AZ-hiPSCs) and from healthy control (ct-hiPSCs). Despite the otherwise comparable levels of hallmark iPCS markers, lower expression level of *SMN1* was found in AZ-hiPSCs compared with control hiPSCs during in vitro primordial germ cell like cells (PGCLCs) differentiation. On the other hand, overexpressing *hSMN1* in AZ-hiPSCs led to increased level of pluripotent markers such as OCT4 and KLF4 during PGCLC differentiation. Our work reveal novel roles of SMN in mammalian spermatogenesis and suggest new therapeutic targets for azoospermia treatment.

## 1. Introduction

In humans, about 15% of couples suffer from infertility, with half of those caused by factors in the male [1,2]. The majority of male infertility is sporadic, due to unexplained abnormalities in sperm parameters, or unexplained azoospermia [3]. For non-obstructive azoospermia (NOA), which defined as no sperm in the ejaculate due to abnormal spermatogenesis, and is the most severe form of male infertility. Unlike the obstructive azoospermia (OA) patients, the etiology of NOA is either intrinsic structure failure of testis or inadequate gonadotropin production, therefore, hormone treatment could improve the testicular function and impaired semen parameters. However, not all patients respond to the hormone treatment [4]. Although numerous genes have proved to have a role during germ cell development in mice, their relevance to human reproduction remains to be clarifying mainly due to the limited access to human tissues and the lack of experimental tools.

Derivation of patient-specific human induced pluripotency stem cells (hiPSCs) by converting the differentiated somatic cells back to pluripotent stage with OSKM factors (*OCT4, SOX2, KLF4 and hc-Myc*,) has become a more important strategy for investigating the cause of developmental diseases. hiPSC is capable of generating almost all kinds of cell and provides unlimited cell source for research, including germ cells [5,6,7]. The system generating germ cell from patient-specific-hiPSC provides a useful tool to study gene functions in germ cell development.

Primordial germ cells (PGCs) originate from postimplantation epiblast cells, which express a transcriptional repressor Blimp1 (PR domain zinc finger protein 1, also known as Prdm1) and show the lineage-restricted characteristics that eventually converts into germline, i.e., sperm and oocyte [8,9]. Surani’s group reported that human ESCs/iPSCs can be derived into human PGC-like cells (hPGCLCs) and found that SOX17 initiates the human PGC specification together with BLIMP1, TFAP2C (Transcription Factor AP-2 Gamma) and Homeobox protein NANOG (NANOG) [10]. Consistently, Sasaki et al. also reported that robust induction hPGCLC from primed hiPSCs via incipient mesoderm-like cells (hiMeLCs) [11]. Unlike the high efficiency of PGCLC induction in human, the generation of haploid cells such as spermatocyte or spermatozoa from the human pluripotent stem cells (hPSCs) encountered obstacles for completing the meiosis process [12,13]. The differentiation procedure can be improved by supplementation with vitamin C, basic fibroblast growth factor (bFGF) and glial cell-derived neurotrophic factor (GDNF) in vitro without mouse testicular somatic cells recently, but the efficiency is still as low as 4 to 5% [13,14].

Recently, our group reported that the levels of survival motor neuron protein (SMN), a major assembler for the process of small nuclear ribonucleoprotein (snRNP) complex, correlate with the capacities of stem cell proliferation and differentiation in *Drosophila* and mice [15,16]. Unlike the main focus of SMN in the neuron degenerative disease, spinal muscular atrophy (SMA) [17,18], we found SMN is highly enriched in the mouse pluripotent ESCs, adult germ cells, and controlling the proliferation and maintenance of spermatogonia [15,19].

In the present work, we hypothesize that SMN participates in spermagogenesis. In support of this, several studies demonstrated that disorder of RNA splicing leads to defects of spermatogenesis [20,21], implying the correct RNA processing facilitates human germ cells development and spermatogenesis. To test this, the mouse spermgatogonia stem cells (SSCs) were isolated and performed in in vitro culture with a SMA-like mouse model (*Smn1^−/−^;SMN2^+^*). Overexpression of SMN sustained the self-renewal of SSCs in wild-type mice, and improved the maturation process of spermatogenesis proven by allogeneic transplant experiment. To further prove the universal phenomenon of SMN in human germ cells, the hiPSCs derived from azoospermia patient (AZ-hiPSCs) was established, characterized, and differentiated. The hPGCLCs were induced from AZ-hiPSC and the SMN expression level was examined throughout this process. Our results provided a practical procedure to investigate potency of the human male germ cell and elucidate the role of SMN in human stem cells.

## 2. Results

### 2.1. Decreased Propagation of Spermatogonia in SMA-Like Mice during In Vitro Culture

**Figure 1:**
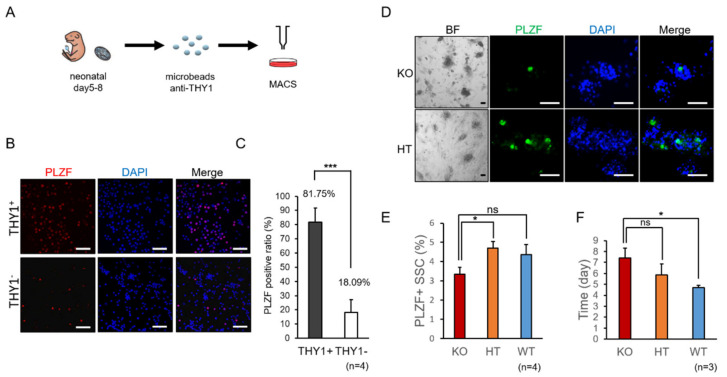
Deficiency of SMN in SSC maintenance in mice. (**A**) Graphic flow chart of SSC isolation. Testis cells for SSCs in vitro culture are obtained from male neonatal pups fractionated by magnetic-activated cell sorting (MACS) with magnetic microbeads conjugated to anti-THY1 (CD 90.2) antibody. (**B**,**C**) Detection of SSC marker PLZF in fractionated SSCs by immunofluorescent staining. 81.75% of PLZF positive cells (red color) is detected in THY1^+^ cells. DAPI is used for DNA stain (blue color). In contrast, fewer (18.09%) THY1^−^ cells presented PLZF (*n* = 3). Scale bar: 200 μm. *t*-test analysis is performed and significant differences is shown (*** *p* < 0.001). (**D**) PLZF expression in SSCs from SMA-like mice after in vitro culture. Bright field (BF) shows the morphology of SSC culture in mouse *Smn1* knockout (KO) and heterozygous littermate control (HT) group. The KO SSCs express less PLZF protein (green color) compared with the HT control. DAPI used for nuclei stain is showed as blue color. Scale bar: 100 μm. (**E**) The percentage of expressing PLZF is quantified in SSCs from KO, HT and wild-type (WT) SMA littermate mice. * Indicates a statistically significant difference, *p* < 0.05. ns indicates no significance.

To determine the specificity for isolating spermatogonia stem cells (SSCs) by Thy-1 Cell Surface Antigen (THY1) through magnetic-activated cell sorting (MACS), the testes of 5–8 days postpartum (dpp) C57BL/6JNarl mice were used and collected as illustrated in Figure 1A. The isolated THY1^+^ SSCs expressed high percentage of spermatogonia marker, promyelocytic leukemia zinc finger (PLZF), also called zinc finger and BTB domain-containing 16 (ZBTB16), detected by immunofluorescent staining (Figure 1B). To identify whether the amount of SMN would affect SSC maintenance, we isolated SSCs from testes of SMA-like mice and their littermate controls at the age of 5–8 dpp for in vitro culture following previously published culture condition [22]. After four passages, SSC derived from SMA pups expressed about 3% of PLZF, which was significantly lower than wild-type and heterozygous littermate controls that possessed over 4% of PLZF positive cells (Figure 1D,E). This data is consistent with our published data, which indicated that loss of SMN affects the maintenance of spermatogonia [19].

### 2.2. Overexpression of SMN1 Promotes Self-Renewal and Homing Ability of Mouse SSCs

**Figure 2:**
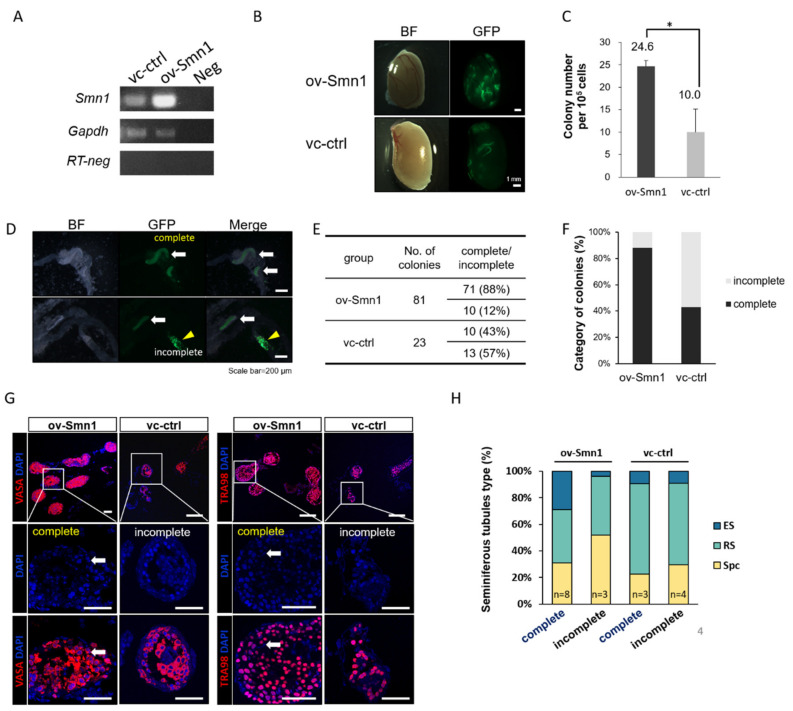
Overexpression of SMN1 promotes self-renewal and homing ability of mouse SSCs. (**A**) abundancy of *Smn1* RNA is confirmed in *Smn1* overexpressed (ov-Smn1) SSCs compared with vector control (vc-ctrl) by semi-quantitative RT-PCR. *Gapdh* is the internal control. RNAs without reverse transcription (RT-neg) is used as the negative control detected by *Gapdh* primer. Neg indicates the non-template control. (**B**) Transplant assay of GFP-SSCs injected into seminiferous tubule in busulfan treat male mice. Bright field (BF) demonstrates the morphology of transplanted testes, and the GFP panel indicates the propagated SSCs in ov-Smn1 and vc-ctrl group. (**C**) Quantification of the efficiency of SSC homing. Triplicates of the transplanted mice shows the significant differences in ov-Smn1 and vc-ctrl group. * Indicates significance, *p* < 0.05. (**D**) Seminiferous tubules with GFP colony shown as complete (white arrow) and incomplete (yellow arrowhead) colonies. (**E**,**F**) Quantification of the ratio of complete and incomplete colonies observed in ov-Smn1 and vc-ctrl group. (**G**) Cross section detecting germ cell markers VASA and TRA98 (red color) in transplanted seminiferous tubules. Complete colonies contain more matured elongated sperms as indicated (white arrow). DAPI is stained for DNA content (Blue). Scale bar: 50 μm. (**H**) Distribution of germ cell types through spermatogenesis in transplant cells. ES: elongated sperm. RS: round spermatids. Spc: spermatocyte.

Next, we focus on the effects of SMN overexpression on mouse SSC. The SMN overexpression lentivirus was transduced into SSC from 5–8 pnd mice carried green fluorescent protein (GFP) (ov-Smn1) and showed increased level of *Smn1* transcripts compared with control vector group (vc-ctrl) analyzed by reverse transcription polymerase chain reaction (RT-PCR) (Figure 2A). SSC transplantation is a critical experimental technique for transfer of germline between donor and recipient males that could be a useful tool for investigating the capacity of donor cells. These *Smn1*-overexpressed GFP-SSCs were allogeneically injected into busulfan treated recipient ICR mice, as well as control vector group. The *Smn1*-overexpressed GFP-SSCs (ov-Smn1) were re-colonized in recipient mice and showed significantly increase ability in colonization compared with vector control (vc-ctrl) (Figure 2B,C). Among these GFP-SSC colonies, more complete colonies were found in ov-Smn1 group than incomplete colonies (88% vs. 12%), indicating the evidence of full spermatogenesis (Figure 2D,E). Unlike ov-Smn1 group, vc-ctrl group only showed about half of the colonies were completely formed (Figure 2E,F). The complete colonies showed general expression of germ cell specific markers, mouse Vasa homolog gene (VASA, also known as DDX4 or MVH) [23,24] and TRA98 [24], and elongated sperms in the cross section, which further confirmed the architecture of spermatogenesis in SSC repopulation both in ov-Smn1 and vc-ctrl group (Figure 2G). The cell type of germ cell was further confirmed with the cell morphology and germ cell marker in cross section. In ov-Smn1 group, we found more elongated sperm cell in complete colony, where lower percentage of elongated sperms were observed in vc-ctrl, indicating the fullness of differentiation capacity in spermatogenesis in ov-Smn1 group (Figure 2H).

### 2.3. Generation and In Vitro Characterization of Human Induced Pluripotent Stem Cells (hiPSCs) from Non-Obstructive Azoospermic Patients

**Figure 3:**
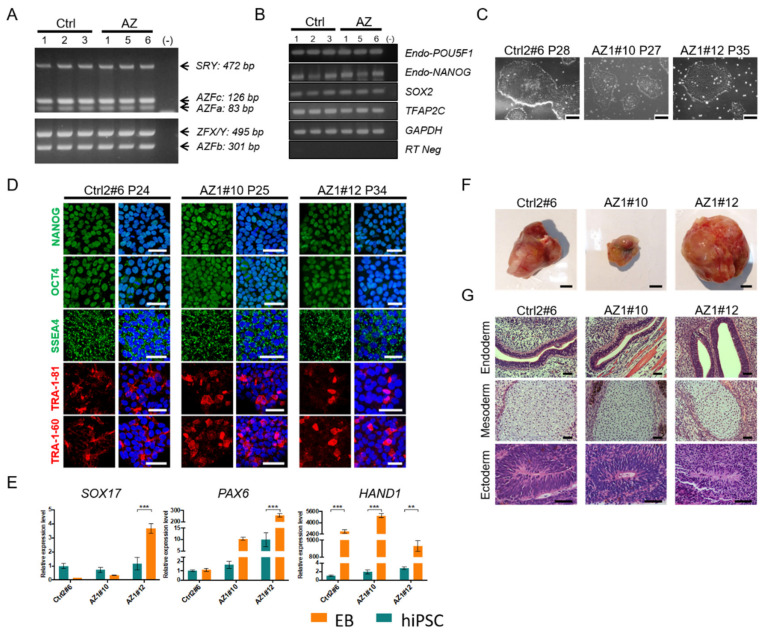
Characterization of the stem cell potency in AZ and control hiPSCs. (**A**) Detection of azoospermia factor (AZF) a, b, and c genes from the genomic DNA of whole blood of azoospermia (AZ1, 2, and 3) and control (Ctrl1, 2 and 3) patients. SRY represents the male specific gene, and ZFX/Y is used as the internal control. (**B**) Pluripotent genes including endogenous *OCT4*, *NAONOG*, *SOX2*, *TFAP2C* are reactivated in hiPSC lines from azoospermia patients (AZ1, 5, and 6) and control men (Ctrl1, 2, and 3). *GAPDH* indicates the internal control, and RT-Neg represents the template RNA without reverse transcription. (−) non template negative control. (**C**) Phase-contrast images of iPSCs derived from control (Ctrl) and 2 azoospermic (AZ) men. Scale bar: 200 μm. (**D**) Immunofluorescence staining of pluripotency-related markers, NANAOG, OCT4, SSEA4, TRA-1-81, and TRA-1-60, in AZ-hiPSC (AZ1#10 and #12) and control (Ctrl2#6) lines. Merged images of pluripotent markers with cell nuclei counter stained with DAPI (blue color). Scale bar: 20 μm. (**E**) Relative expression levels of germlayer specific genes (*SOX17* for endoderm, *PAX6* for ectoderm, and *HAND1* for mesoderm) in hiPSCs and 10-day EBs analyzed by real-time RT-PCR. Error bars indicate mean ± SEM from three replicates (two-way ANOVA, ** *p* < 0.01; *** *p* < 0.001). (**F**) Morphology of teratoma tissue in AZ-hiPSC lines (AZ1#10 and #12) and control hiPSCs (Ctrl2#6) collected from NOD/SCID mice. Scale bar: 5 mm. (**G**) Representative images of H&E-stained teratoma section from hiPSCs demonstrate the potency of three germ layers. Ciliated cells (endoderm), cartilage-like cells (mesoderm) and primitive neuronal cells (ectoderm) can be observed in all three hiPSC lines. Scale bar: 50 μm.

Establishing NOA patient-specific hiPSCs offers a promising tool for studying male infertility and developing new therapeutic options for treatment. To further investigate whether SMN overexpression also facilitate the process of spermatogenesis in human, we established the hiPSCs from NOA patients (AZ-hiPSCs) by using Sendai virus to transduce the reprogramming factors, OCT4, SOX2, KLF4 and hc-MYC (OSKM) into peripheral blood mononuclear cells (PBMCs) that isolated from NOA patients’ blood. We identified three NOA patients who were identified as azoospermia factor (AZF) non-relative azoospermia which the AZF genes were detected normally as the control man (Figure 3A). We generated patient-specific azoospermia iPSC lines (AZ1, 5 and 6) from three azoospermia patients, and from three healthy control individuals (Ctrl 1, 2, and 3). All these AZ and control iPSC lines expressed endogenous pluripotent markers detected by RT-PCR analysis (Figure 3B). Control individual 2 sub-clone (Ctrl2#6) and sub-clones (AZ1#10, AZ1#12) from the AZ1 patient which were histologically diagnosed as hypospermatogenesis in testis were selected for further culture and showed consistent morphology at later passages and sustained pluripotent markers detected by immunofluorescent staining (Figure 3C,D). Furthermore, we confirmed the absence of Sendai virus genome and transgenes in all hiPSC lines except for a small amount of Sendai virus genome was detected in clone Ctrl2#6 (Supplementary Figure S1). Next, the differentiation potency of three hiPSC clones was explored by EB formation and teratoma assay. Real-time RT-PCR demonstrated the differentiation markers of *SOX17*, *PAX6* and *HAND1* were up-regulated upon differentiation in those hiPSC lines, but no significant changes in the expression of SOX17 in Ctrl2#6 and AZ1#10 (Figure 3E). Histological analysis of teratoma derived from clone Ctrl2#6, AZ1#10, and AZ1#12 reveals their capacity of differentiating into endoderm, mesoderm, and ectoderm (Figure 3F,G). Hence, the expression of typical pluripotency-related genes and differentiation capacity of three germ layers in these hiPSCs derived from both healthy and NOA patients suggest the successful reprogramming in those hiPSC lines.

### 2.4. Induction of Human Primordial Germ Cell-Like Cells (hPGCLCs) in SMN Overexpressed Azoospermia hiPSCs 

**Figure 4:**
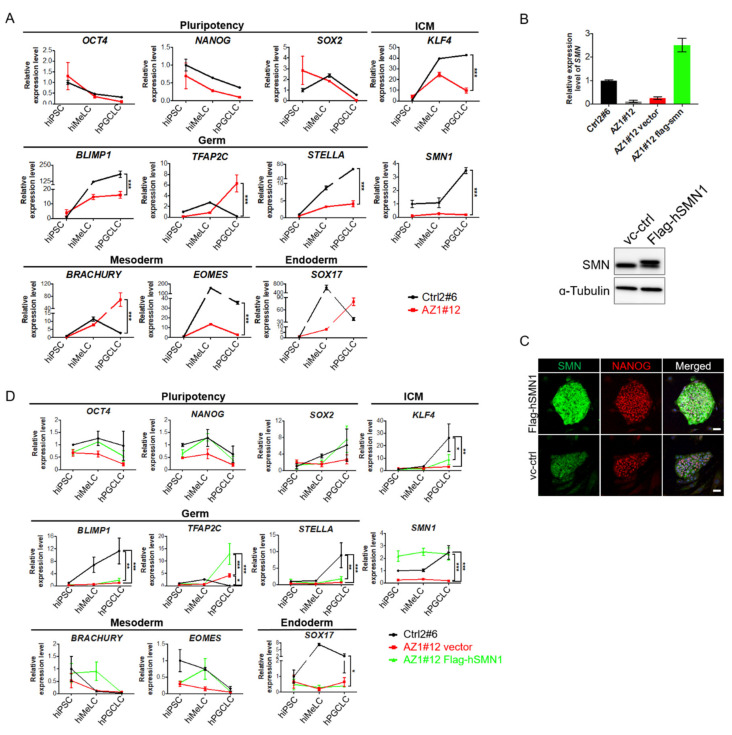
Effect of *hSMN1* overexpression on hPGCLC induction in hiPSC from azoospermia patients. (**A**) Expression analysis by real-time RT-PCR of hiPSCs, hiMeLCs, and hPGCLCs. Pluripotent markers including *OCT4*, *NANOG* and *SOX2*, as well as ICM marker *KLF4* are down-regulated upon differentiation, whereas germ cell markers such as *BLIMP1*, *TFAP2C,* and *STELLA* are up-regulated during differentiation in control hiPSC (Ctrl2#6). Mesoderm (*BRACHYURY*, *EOMES*) and endoderm marker (*SOX17*) show the increasing tendency during differentiation. *SMN1* expresses abundantly in cells from Ctrl2#6 hiPSCs compared with AZ1#12 group. Relative expression levels are shown with normalization to *GAPDH*. Error bars indicate mean ± SEM from three replicates (two-way ANOVA, * *p* < 0.05; ** *p* < 0.01; *** *p* < 0.001). (**B**) Comparison of *SMN1* transcripts (upper panel) in Ctrl2#6 and AZ1#12 hiPSC by real-time RT-PCR. Relative fold change of *SMN1* is significantly elevated in Flag-hSMN1 overexpressed AZ-hiPSCs (Flag-hSMN1) compared with vector control (vc-ctrl). Western blot analysis demonstrates the exogeneous Flag-tagged SMN protein in SMN overexpressed group (Flag-hSMN1) in 4i culture condition. α-Tubulin is used as internal control (lower panel). (**C**) Immunofluorescent staining of Flag-hSMN1 overexpressed AZ-hiPSCs (Flag-hSMN1) in 4i condition (left panel). Compared with vector control (vc-ctrl), abundancy of SMN (green) is shown in Flag-hSMN1 group. NANOG (red) is stably expressed in those hiPSC lines. Scale bar: 50 μm. (**C**) Rela-time PCR analysis on Flag-hSMN1 overexpressed AZ-hiPSCs from stages of hiPSCs, hiMeLC to of hPGCLC induction. Markers associated with pluripotency, germ cell development, mesoderm and endoderm differentiation are examined on hPGCLC from control hiPSCs (Ctrl2#6), AZ-hiPSCs introduced with control vector (AZ1#12 vector) and Flag-hSMN1 vector (AZ1#12 Flag-hSMN1).

As the precursors of oocyte and sperm, PGCs are the earliest embryonic progenitors in the germline. We wonder if patient-specific male gametes could be produced through in vitro hPGCLCS induction from AZ-hiPSCs. Unlike in mice, human PGCLCs must be derived via an incipient mesoderm-like cell (hiMeLCs) state from hiPSCs [11,25,26]. Therefore, the hiMeLCs were induced for three days from 4i medium adapted hiPSC lines following previous published protocol [10] and then hPGCLC aggregates were formed for four days. Mesoderm- and endoderm-relative genes such as *EOMES* and *SOX17* were up-regulated in iMeLC condition, and the PGC markers such as *BLIMP1* and *STELLA* were significantly increased upon induction in the derivatives from Ctrl2#6 compared with the pluripotent status by real-time RT-PCR analysis (Figure 4A). It is worth noting that the expression level of *SMN1* was significantly lower in the AZ cells compared with control cells throughout 4i culture condition till PGCLC stages (Figure 4A).

Considering the function of SMN in spermatogenesis and pluripotency maintenance, we generated the Flag-tagged SMN-continuously expressed AZ-hiPSC lines from AZ1#12 (Flag-hSMN1) by lentiviral transduction to explore the benefits of SMN in differentiation capacity from hiPSCs to hPGCLCs. The real-time RT-PCR and western blot analysis indicated the abundance of SMN and exogenous Flag expression in Flag-hSMN1 hiPSC line compared with the vector control group (Figure 4B). The pluripotent marker NANOG was stably expressed in the Flag-hSMN1 AZ-hiPSCs, indicating the unaffected pluripotency upon exogenous gene transduction (Figure 4C). These Flag-hSMN1 AZ-hiPSCs were differentiated into PGCLCs to investigate whether overexpression of SMN could facilitate the germ cell differentiation. To our surprise, the pluripotent markers, *OCT4*, *SOX2*, and *NANOG,* were significantly increased upon SMN transduction at PGC stage, whereas the PGC and mesoderm markers were relatively stable during differentiation (Figure 4C).

## 3. Discussion

Previously, our group demonstrated that the deficiency of SMN results in the defects on pluripotent stem cells, affecting neurogenesis and spermatogenesis in mice [15,19]. Although SMN serves as a housekeeping gene in various tissues and types of cells, how SMN affects stem cells still need to be elucidated. In this study, we analyzed the effect of SMN on mouse spermatogenesis and human germ cell differentiation. Using an in vitro culture assay, we found the SMN deficient SSCs were unstable during maintenance and loss of spermatogonia marker PLZF after continuous culture (Figure 1), which is consistent with our previous in vivo study [19].

Previously, we reported that overexpression of SMN in mouse ESC sustained its undifferentiation status and showed higher potency in resistance of retinoid acid induced differentiation [15]. Here, similar phenomenon also showed in mouse SSCs. Importantly, upon allogeneic transplant experiments, Smn1-overexpressed SSCs demonstrated significantly increased homing ability. The molecular mechanism remains unclear. Given that SMN serves as the key assembler in RNA splicing process and responsible for resolving the RNA:DNA hybrids, R-loop, and subsequently anti-apoptosis [7,27,28], it is reasonable to speculate that increased level of SMN could speed up the RNA processing and sustained the cell survival in SSCs, therefore resulted in the advanced GFP colonies (Figure 2).

In the present work, We followed the long-turn culture condition of mouse SSC reported recent years, including (1) StemPro-based medium, (2) supplementation with growth factor cocktail of GDNF and bFGF, (3) co-culture with growth inactivated MEF feeder cells, and (4) maintenance in an atmosphere of 5% CO_2_ and 21% O_2_ tension [22]. Although cultured SSCs expressed spermatogonia marker PLZF, heterogeneous differentiated spermatogonial populations still massively retained under our long-turn culture. Differences of purification procedure could affect the component when extraction of bovine serum albumin (BSA), soluble lipids and conformation of albumin both contribute to SSC maintenance [22,29]. Improvement of culture condition should be considered in future studies.

We generated several AZ-hiPSC lines by the integration-free method and serum free culture condition. These hiPSCs showed characteristics of pluripotent stem cells and capable for three germ layer differentiation (Figure 3). The germ cell differentiation was examined in those AZ-hiPSC lines and they showed low level of PGC markers, *BLIMP1* and *STELLA* compared with control group, as well as *SMN1* (Figure 4). To our knowledge, this is the first report studying in the correlation of SMN and spermatogenesis of azoospermia patients. This finding corroborates a recent genome-wide analysis which demonstrated that the association of azoospermia, and incorrect splicing of mRNAs [30]. Because deficiency of SMN mainly leads to the failure of mRNA process, our study provides a concept of potential therapy for NOA patients with abnormal splicing mRNA using the clinically applicable drug as used in SMA patients. [18,31]. Various clinical SMA drug has been approved recent years by the Food and Drug Administration (FDA), such as antisense oligo nucleotides (ASOs) drug Nusinersen (Spinraza) by correction of SMN2 exon 7 splicing, gene therapy targeting *SMN1* using AAV [31], and an orally deliverable small molecule drug-risdiplam (Evrysdi) that the therapeutic effect would be able to reach to all organs [32]. In this regard, augmenting SMN levels may prove to be a viable strategy to rescue/improve the spermatogenesis efficiencies ex vivo or in vivo for NOA patients.

Together, our work demonstrates that SMN plays important roles in spermatogenesis in mouse and in human, and suggests new therapeutic targets for treating azoospermia.

## 4. Materials and Methods

### 4.1. Availability of Data and Materials

The work was approved by Institutional Review Boards (IRB) at Mackay Memorial Hospital (MMH) (IRB approval number:16MMHIS178e). The animal maintenance, care, and procedures described within were reviewed and approved by the Institutional Animal Care and Use Committee of National Taiwan University (NTU) according to the protocol number (NTU-107-EL-154, 11th January 2018 approved, NTU-108-EL-174, 11th May 2020 approved). All methods in the manuscript were performed in accordance with the relevant guidelines and regulations of NTU. Graphics and tables in the manuscript were prepared by the first and corresponding authors. Unless indicated, all reagents were purchased from Thermo Fisher Scientific company (Waltham, MA, USA).

### 4.2. Generation and Culture of hiPSCs from Azoospermic Patients

The key reprogramming factors hOCT, hKLF4, hSOX2, and hC-MYC (OSKM) were introduced by non-integrating Sendai virus by using CytoTune-iPS 2.0 Sendai Reprogramming Kit (A16517) following manufacturer’s instruction. Briefly, the peripheral blood monocyte cells (PBMCs) from azoospermia patients or control individuals were isolated by Ficoll-Paque PLUS (GE17-1440-02, Sigma, St. Louis, MO, USA) from whole blood and freeze immediately until usage. PBMCs were plated in complete StemPro-34 (10639-011) medium containing the cytokines, such as SCF (c-kit Ligand), FLT-3 Ligand, IL-3, IL-6, and GM-CSF (PHC2111, PHC9414, PHC0034, PHC0065) for four days and the infected with Sendai virus carrying OSKM for one day. After infection, cytokines were removed from StemPro-34 and cultured for 3 days. Hereafter, cells were transferred to Matrigel-coated plates (354234, BD Biosciences, San Jose, CA, USA) and maintained in the StemFlex Medium (A3349401). The estimative time for colony forming were 9 to 28 days after infection. The hiPSC-like colonies were manually isolated based on morphology between Day 21 to Day 27 post-transduction and cultured as iPSCs hereafter. For routine passage, TrypLE express (12605010) was used to dissociate colonies every 4–5 days and cells were replated on Matrigel-coated plates in StemFlex medium. All cells were cultured at 37 °C in a humidified atmosphere containing 5% CO_2_.

### 4.3. Transduction of SMN into hiPSCs or Mouse SSCs with Lentivirus

The lentiviral mouse *Smn1* expressing vector activated by EF1 alpha promoter was constructed from the pSin-EF2 plasmid (#16578, addgene, Watertown, MA, USA) by replacing NANOG gene with *Spe*I and *BamH*I. Human *SMN1* carrying flag sequence was replaced into the same plasmid through *Spe*I and *EcoR*I cutting site. Inserted genes were amplified from cDNA of mouse testis or hciPSCs by HiFi PCR Kit (KR0368, KAPA Biosystems, Wilmington, MA, USA) using specific primers listed in Supplementary Table S1. Packaging and envelop vectors from RNAi core (Academia Sinica, Taipei, Taiwan) were transfected together with lentiviral expressing vector into 293T cells to produce Flag-hSMN1 lentivirus. TrypLE express-dissociated single hiPSCs were infected in suspension with SMN-lentivirus for one day in Metrigel coated plates. On the second day, the virus was removed and changed to fresh StemFlex medium until colony regrows to 0.5 mm diameter.

### 4.4. Induction of hiMeLCs, hPGCLC

Prior to hiMeLCs and hPGCLC induction, control hiPSC (Ctrl2#6) and azoospermia iPSC (AZ1#12) were cultured in 4i hESM medium for 10 passages. The 4i hESM is composed of 20% Knockout Serum Replacement (KSR, 10828028) in Knockout DMEM (10829018), with the following additions: 3 μM CHIR99021 (Axon 1368. Axon Medchem BV, Groningen, The Netherlands), 1 μM PD0325901 (Axon 1408, Axon), 5 μM SB203580 (1202, Tocris Bioscience, QL, UK), and 5 μM SP600125 (1496, Tocris), Activin A (20 ng/mL, Peprotech, Rocky Hill, NJ, USA), human LIF (20 ng/mL, 300-05, Peprotech), and a higher concentration of bFGF (8 ng/mL) as previou published protocol [10]. For iMeLC induction, 4i adapted hiPSC were plated onto a human plasma fibronectin (33016015)-coated 12-well plate at 2 × 10^5^/well in DMEM/F12 (11330–032) medium supplemented with 1% KSR, 1X N2 supplement (17502–048), 1X B27 supplement (17504–044), basic fibroblast growth factor (bFGF, 10 ng/mL, 13256029), Activin A (120–14P, 20 ng/mL, PeproTech, Rocky Hill, NJ), and ROCK inhibitor Y27632 (10 μM, 1254, Tocris Bioscience, Bristol, UK) for 2 days. The hPGCLCs were induced by plating 2000–4000 TrypLE express-singlets of hiMeLCs into a ultra-low attachment U-bottom 96-well plate (174925) in Glasgow’s MEM (GMEM, 11710035) supplemented with nonessential amino acids (0.1 mM, NEAA, 11140–050), 2-mercaptoethanol (0.1 mM, ES–007–E, Millipore, Burlington, MA, USA), GlutaMax (2 mM, 35050–061), 1X Penicillin and Streptomycin (P/S, 15070–063), sodium pyruvate (1 mM, 11360–070), BMP4 (250 ng/mL, 314–BP–050, R&D, Minneapolis, MN, USA), BMP2 (355–BM–050, 250 ng/mL, R&D), human LIF (20 ng/mL, 300–05, Peprotech), stem cell factor (SCF, 100 ng/mL, PHC2115), epidermal growth factor (EGF, 50 ng/mL, PHG0313), and 10 μM of Y27632 for 4 days.

### 4.5. In Vitro Proliferation of Mouse SSCs

Mouse SSCs were isolated from the testis of six to seven days postpartum (dpp) C57BL/6JNarl or SMA-like mice [C57BL/6/*Tg(SMN2) Hung Smn1^tm1 Hung^*] (National Laboratory Animal Center, NLAC, Taipei, Taiwan) [33]. After Collagenase IV and Trypsin digestion, testicle cells were incubated with anti-CD90.2 (THY1) antibody and then labeled with anti-biotin microbeads (130-090-485, Miltenyi Biotec, Bergisch Gladbach, Germany). 2 × 10^5^ SSCs were cultured in gelatin-coated 24-well plate seed with mitomycin C treated mouse embryonic fibroblast (MEF) feeders and maintained in SSC medium composed of complete StemPro-34 medium (10639011) supplemented with 5 mg/mL of BSA (A4378, Sigma), 1% of ES cell-qualified heat-inactivated FBS (16000-044), D-(+)-glucose (6 mg/mL, G6152, Sigma), 2-mercaptoethanol, ITS, P/S, GlutaMax, NEAA, MEM vitamin solution (11120052), 0.14% (wt/vol) of sodium bicarbonate (S5761, Sigma), sodium selenite (30 nM, S5261, Sigma), sodium pyruvate (30 μg/mL, P3662, Sigma), Putrescine (60 μM, P7630, Sigma), ascorbic acid (100 nM, A4403, Sigma), D-biotin (10 μg/mL, B4639, Sigma), progesterone (60 ng/mL, P0130, Sigma), β-estradiol (30 ng/mL, E88775, Sigma), EGF (20 ng/mL), GDNF (10 ng/mL), and bFGF (10 ng/mL). Half of the medium were changed daily till confluence and passed by TrypLE to new feeder for further culture.

### 4.6. Immunofluorescent Staining and Confocal Microscopy

Cells were fixed with 4% paraformaldehyde (PFA) in DPBS for 20 min. Fixed cells were incubated with the primary antibody in PBS with 2.5% bovine serum albumin and 0.25% Triton-X100 for one hours at room temperature and incubate with primary antibody at 4 °C for overnight. After washing with PBS, samples were incubated with secondary antibody, which is diluted in the same solution as used in primary antibody dilution. One hours after the secondary antibody reaction, samples were washed and mounted with ProLong Antifade Mountanting medium (P36984). Primary antibodies used in this study include: NANOG (1:500, ab21624, Abcam, Boston, MA, USA), OCT4 (1:150, MAB4401, Millipore), SOX2 (1:150, GTX101507, Genetex, Alton Pkwy Irvine, CA, USA), SSEA4 (1:150, MAB4304, Millipore), TRA1-60 (1:200, MAB4360, Millipore), TRA-1-81 (1:200, MAB4381, Millipore). Secondary antibodies (4 μg/mL) were listed as bellow: Alexa Fluor goat anti-mouse 488 and 546 (A11029 and A10036), goat anti-mouse IgM 594 (A21044), goat anti-rabbit 488 (A32731). Images were acquired with confocal microscope (TCS SP5 II, Leica, Wetzlar, Germany).

### 4.7. Immunohistochemistry (IHC)

Testis tissues of mice were dissected and immersed in 10% Formalin for overnight at 4 °C, then embedded into wax. Sections were dewaxed and treated with antigen retrieval solution (HK057-5K, BioGenex, Fremont, CA, USA) as manufacture’s guide. The germ cell markers, PLZF (1:100, sc–28319, Santa Cruz Biotechnology Inc., Dallas, CA, USA), rat anti-TRA98 (1:150, ab82527, Abcam) and VASA (1:1000, ab13840, Abcam) were stained in testis sections. Isotype IgG including rabbit (550875, BD) and rat (559072, BD) were used as the negative control as shown in Supplementary Figure S2. Secondary antibodies (1:500) were used as follows: Alexa Fluor donkey anti-rat Cy5(A10525) and Alexa Fluor goat anti-rabbit IgG 594 (A11012).

### 4.8. Western Blotting

Cell lysates from tissues (30 μg/lane) were run on a 12% polyacrylamide gel and then transferred to PVDF membrane. Following 1 h incubation for blocking non-specific binding with 5% non-fat dried milk in TBS, first antibody was incubated overnight at 4 °C including mouse anti-SMN antibody (1:20,000) and α-Tubulin (1:20,000, T5168, Sigma) was used as an internal control. After several washes containing 0.1% Tween-20 in TBS, the blot was incubated for 1 h with a HRP-conjugated goat anti-mouse secondary antibody (31,460). The bound antibody was detected by GeneGnome XRQ chemiluminescence detection system (Cambridge, UK).

### 4.9. Gene Expression Analysis

Total RNA was isolated by TRIzol^®^ reagent (15596026) and treated with RNase-free DNase I (M6101, Promega, Madison, WI, USA) to remove genomic DNA. Treated RNAs were reverse-transcribed by random hexamer primers using the GoScript Reverse Transcription System (A2801, Promega). cDNA (25 ng) was mixed with Hieff qPCR SYBR Green Master Mix (11203ES03, Shanghai, China) and 200 nM of the forward/reverse primers in a final volume of 10 μL. Real-time RT PCR was performed using the Roche LightCycler (LC480, Roche Applied Science, Mannhein, Germany), 15 s at 95 °C and 30 s at 60 °C for 45 cycles, and followed by the thermal denaturing step to generate the dissociation curves to verify amplification specificity. All the genes were normalized with the CT value of GAPDH. For conventional semi-quantitative PCR, cDNA (50 ng) was assayed as 30 s at 95 °C, 30 s at 55 °C and 30 s at 72 °C for 35 cycles by PCR machine (Biorad). PCR products were run on agarose gel electrophoresis and photographed by gel image system (UVP). The primer sequences were listed as in Supplementary Table S2.

### 4.10. Teratoma Assay

hiPSCs were dissociated by TrypLE and suspended in Matrigel/DMEM mixture. At least 5 × 10^6^ were injected intramuscularly into a 6–8-week-old NOD/SCID mouse (National Laboratory Animal Center, NLAC, Taipei, Taiwan). Teratomas were collected 6 weeks post-transplantation. Teratoma tissues were dissected and immersed in 10% formaldehyde overnight at 4 °C and then embedded into wax. Sections were dewaxed, rehydrated, and stained with hematoxylin and Eosin (H&E).

### 4.11. Germ cell Transplantation

β-Actin promoter-driven GFP (GFP) 5–8 dpp ICR mice backcross with C57BL/6JNarl for 20 generations and were used for donor mouse SSCs in the germ cell transplantation experiment [33,34]. Six week old recipient ICR male mice (BioLASCO, Taipei, Taiwan) were treated with 40 mg/kg busulfan (B2635, Sigma) and kept for one month to eliminate endogenous germ cells [22,29]. Following previously described procedures [19,35], the testicle THY1^+^ SSC cell suspension from 5–8 dpp GFP mice (concentration of 2.5 × 10^6^ cells/mL) was mixed with Trypan Blue dye (T8154, Sigma) in Polyvinyl alcohol (PVA, P8136, Sigma) containing D-PBS solution, and approximately 20 μL of the cell suspension was injected through efferent duct into rete testis of the recipients. Two months later, the testes of the recipient animals were dissected for detecting of GFP^+^ colonies.

### 4.12. Statistics

All data were presented as means ± standard error of the mean (SEM) and unpaired comparison was analyzed using the Student’s *t*-test or one-way ANOVA with Dunnett’s multiple comparison test (GraphPad Software Inc., La Jolla, CA, USA). Significance was assumed at a *p*-value of 0.05.

## 5. Conclusions

In this study, we demonstrated that defected SMN results in loss of spermatogonia in in vitro culture. In contrast, increased level of SMN not only promotes self-renewal and homing ability in mouse SSCs, but also elevated the colony completeness of seminiferous tubules in allogeneic transplant assay. The hiPSCs derived from azoospermia patient showed the differentiation ability in vitro and in vivo. AZ-hiPSCs expressed all pluripotent markers during long term in vitro culture, and typical cell types with three germlayers were featured in the teratoma assay, indicating the successful reprogramming of these hiPSC lines. The developmental capacity of germ cell lineage was assayed by the hPGCLC induction procedures. The decreased *SMN1* expression pattern during the differentiation process from hiPSCs, hiMeLCs, and hPGCLCs in AZ-hiPSC, implying that the defected expression of SMN might be one of the potential factors that cause of azoospermia. When overexpressing *hSMN1* in AZ-hiPSCs, pluripotent markers expressed at increased tendency compared to the control vector group. However, the PGC related genes could not be elevated in these hPGCLCs, implying the concealed defect beyond SMN might still exist in examined AZ cells. In summary, current study showed that SMN can improve the propagation of SSCs, and patient-specific hiPSCs can be generated from azoospermia patients, which provides a potential tool for artificial gamete production to investigate male infertility and explore novel therapy in the future.

## Data Availability

The data presented in this study are available in the article and supplementary data.

## Supplementary Materials

Supplementary Materials can be found at https://www.mdpi.com/1422-0067/22/2/661/s1.
