# Supplementary material for: Effects of Survival Motor Neuron Protein on Germ Cell Development in Mouse and Human
Source: Int J Mol Sci. 2021 Jan 11;22(2):661. doi: 10.3390/ijms22020661 (PMC7827477; doi:10.3390/ijms22020661)
Supplement: Supplementary file 1 [file ijms-22-00661-s001.pdf]

Effects of Survival Motor Neuron Protein on Germ Cell development in Mouse and Human

Wei-Fang Chang, Min Peng, Jing Hsu, Jie Xu, Huan-Chieh Cho, Hsiu-Mei Hsieh-Li, Ji-Long Liu, Chung-Hao Lu, and Li-Ying Sung

SUPPLEMENTAL INFORMATION

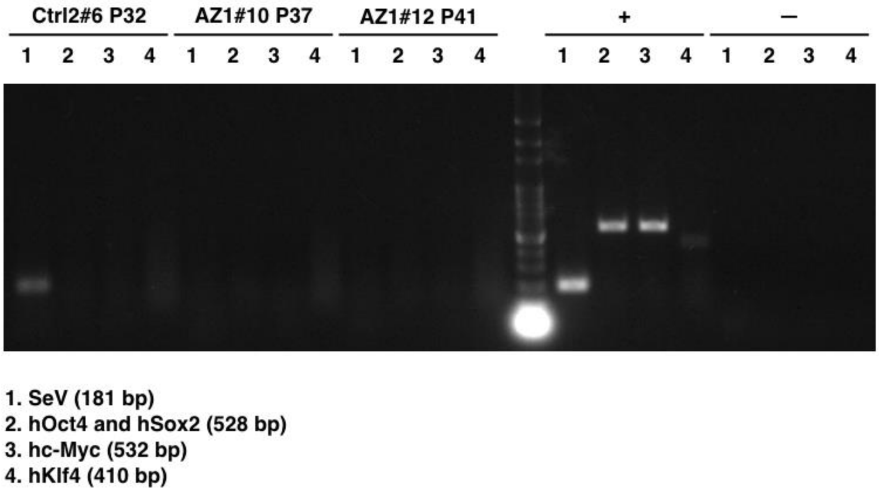

Supplementary Figure S1. Sendai virus detection of hiPSC lines by semin-quantitative RT-PCR. All hiPSC lines shows no exogenous OSKM expression, except the sendai virus genome was weakly detected in Ctrl2#6 hiPSC line. +: mouse lung cells infected OSKM Sendai virus. -: water without template as negative control.

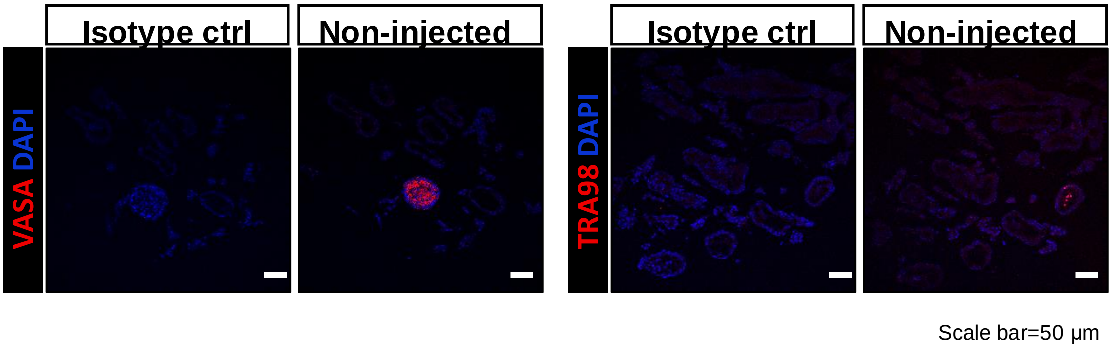

Supplementary Figure S2. Validation of antibody specificity in IHC. The testis of ICR mice treated with busulfan for 4 months was used as the control for antibody detection.

Table S1. Primers for plasmid construction

5`SpeI-hSMN1	5′-GACTAGTCATGGCGATGAGCAGCGGCGGCAGTGGTGGC-3′
3`EcoRI-hSMN1	5′-GGAATTCCTTAATTTAAGGAATGTGAGCACCTTCCTTCTTTTTGAT-3′
5' SpeI-SMN(ms)	5′-GACTAGTCGTCATTGAGTGAGCCCGGCAGC-3′
3`BamHI-SMN(ms)	5′-CGGGATCCCTCCTGAGACAGAGCTGAACTTCTTA-3′

Table S2. Primer sequences used for real-time RT-PCR.

Targeting gene	Primer sequences
hZFY	5′-ACCRCTGTACTGACTGTGATTACAC-3′ and 5′-GCACYTCTTTGGTATCYGAGAAAGT-3′
hSRY	5′-GAATATTCCCGCTCTCCGGA-3′ and 5′-GCTGGTGCTCCATTCTTGAG-3′
G34990(hAZFa)	5′-CATTCGGTTTTATCAGCCAG-3′ and 5′-CAGTGACTCGAGGTTCAATG-3′
sY134(hAZFb)	5′-GTCTGCCTCACCATAAAACG-3′ and 5′-ACCACTGCCAAAACTTTCAA-3′
sY255(hAZFc)	5′-GTTACAGGATTCGGCGTGAT-3′ and 5′-CTCGTCATGTGCAGCCAC-3′
mSmn1	5′-GCTCC GTGGA CCTCA TTTC-3′ and 5′- GGGCC GTTGA ATTTT AGACC-3′
mGapdh	5′-CCCTTCATTGACCTCAACTA-3′ and 5′-CCAAAGTTGTCATGGATGAC-3′
SeV genome	5′-GGATCACTAGGTGATATCGAGC-3′ and 5′-ACCAGACAAGAGTTTAAGAGATATGTATC-3′
Exogenous Sendai hKOS	5′-ATGCACCGCTACGACGTGAGCGC-3′ and 5′-ACCTTGACAATCCTGATGTGG-3′
Exogenous Sendai KLF4	5′-TTCCTGCATGCCAGAGGA-3′ and 5′-AATGTATCGAAGGTGCTCAACC-3′
Exogenous Sendai hcMyc	5′-TAACTGACTAGCAGGCTTGTCG-3′ and 5′-TCCACATACAGTCCTGGATGATG-3′
hOCT4	5′-AGTTTGTGCCAGGGTTTTTG-3′ and 5′-ACTTCACCTTCCCTCCAACC-3′
hNANOG	5′-TTTGGAAGCTGCTGGGGAAG-3′ and 5′-GATGGGAGGAGGGGAGAGGA-3′
hSOX2_2	5′-GCGATGCCGACAAGAAAACT-3′ and 5′- ACTTCCTGCAAAGCTCCTACC-3′
hEOMES	5′-CTGGCTTCCGTGCCCACGTC-3′ and 5′- CATGCGCCTGCCCTGTTTCG-3′
hKLF4	5′-TACCAAGAGCTCATGCCACC-3′ and 5′- CGCGTAATCACAAGTGTGGG-3′
hSTELLA	5′-ACGCCGATGGACCCATCACAGTTT-3′ and 5′-TCTCGGAGGAGATTTGAGAGGCCC-3′
hBRACHURY	5′-ACCCAGTTCATAGCGGTGAC-3′ and 5′-CCATTGGGAGTACCCAGGT-3′
hSOX17	5′-ACGCCGAGTTGAGCAAGA-3′ and 5′-GTGCAGGAAGCCGCCCTC-3′
hBLIMP1	5′-CAGTGTTGCGGAGAGGCAAG-3′ and 5′-TCTGCCAATCCCTGAAACCTC-3′
TFAP2C	5′-CGCTCATGTGACTCTCCTGACATCC-3′ and 5′-

	TGGGCCGCCAATAGCATGTTCT-3′
hGAPDH	5′-AGGGCTGCTTTTAACTCTGGT-3′ and 5′- CCCCACTTGATTTTGGAGGGA-3′
